# The Weissella Genus: Clinically Treatable Bacteria with Antimicrobial/Probiotic Effects on Inflammation and Cancer

**DOI:** 10.3390/microorganisms10122427

**Published:** 2022-12-07

**Authors:** Sadia Ahmed, Sargun Singh, Vaidhvi Singh, Kyle D. Roberts, Arsalan Zaidi, Alexander Rodriguez-Palacios

**Affiliations:** 1 Division of Gastroenterology and Liver Disease, Case Western Reserve University School of Medicine, Cleveland, OH 44106, USA; 2 Digestive Health Research Institute, Case Western Reserve University School of Medicine, Cleveland, OH 44106, USA; 3 National Probiotic Laboratory, National Institute for Biotechnology and Genetic Engineering (NIBGE-C), Faisalabad 38000, Pakistan; 4 Pakistan Institute of Engineering and Applied Sciences, Nilore, Islamabad 45650, Pakistan; 5 University Hospitals Research and Education Institute, University Hospitals Cleveland Medical Center, Cleveland, OH 44106, USA; 6 Department of Molecular Biology and Microbiology, Case Western Reserve University School of Medicine, Cleveland, OH 44106, USA

**Keywords:** probiotic, antimicrobial, anti-inflammatory, anticancer, GRAS, starter culture, food, gut

## Abstract

*Weissella* is a genus earlier considered a member of the family *Leuconostocaceae,* which was reclassified into the family *Lactobacillaceae* in 1993. Recently, there have been studies emphasizing the probiotic and anti-inflammatory potential of various species of *Weissella*, of which *W. confusa* and *W. cibaria* are the most representative. Other species within this genus include: *W. paramesenteroides, W. viridescens, W. halotolerans, W. minor, W. kandleri, W. soli, W. ghanensis, W. hellenica, W. thailandensis, W. fabalis, W. cryptocerci, W. koreensis, W. beninensis, W. fabaria, W. oryzae, W. ceti, W. uvarum, W. bombi, W. sagaensis, W. kimchi, W. muntiaci, W. jogaejeotgali, W. coleopterorum, W. hanii, W. salipiscis,* and *W. diestrammenae. Weissella confusa, W. paramesenteroides, W. koreensis,* and *W. cibaria* are among the few species that have been isolated from human samples, although the identification of these and other species is possible using metagenomics, as we have shown for inflammatory bowel disease (IBD) and healthy controls. We were able to isolate *Weissella* in gut-associated bacteria (post 24 h food deprivation and laxatives). Other sources of isolation include fermented food, soil, and skin/gut/saliva of insects/animals. With the potential for hospital and industrial applications, there is a concern about possible infections. Herein, we present the current applications of *Weissella* on its antimicrobial and anti-inflammatory mechanistic effects, the predisposing factors (e.g., vancomycin) for pathogenicity in humans, and the antimicrobials used in patients. To address the medical concerns, we examined 28 case reports focused on *W. confusa* and found that 78.5% of infections were bacteremia (of which 7 were fatal; 1 for lack of treatment), 8 were associated with underlying malignancies, and 8 with gastrointestinal procedures/diseases of which 2 were Crohn’s disease patients. In cases of a successful resolution, commonly administered antibiotics included: cephalosporin, ampicillin, piperacillin-tazobactam, and daptomycin. Despite reports of *Weissella*-related infections, the evolving mechanistic findings suggest that *Weissella* are clinically treatable bacteria with emerging antimicrobial and probiotic benefits ranging from oral health, skin care, obesity, and inflammatory diseases to cancer.

## 1. Introduction

The *Weissella* genus has begun to take center stage in the past few years owing to its probiotic potential and its many prospective applications, ranging from the healthcare industry to the skin care and food industries. Due to its ability to thrive in stomach acid and bile, adherence to intestinal cells, and its antimicrobial potential against other pathogenic microorganisms including but not limited to *Staphylococcus aureus*, *Listeria monocytogenes*, *Salmonella typhi,* and *Salmonella enterica*, most *Weissella* species meet the pre-requisites needed to be classified as a probiotic. The only limitation to its widespread use is the lack of a significant volume of research at the moment and a handful of reported cases of pathogenicity. However, a bulk of these cases are a result of some preexisting disposition or comorbidity associated with the host. Despite such pathogenic potential, we set to investigate to what extent this genus is clinically treatable with common antimicrobials in the event of its identification in human infections. Herein, we primarily describe the antimicrobial and anti-inflammatory potential of *Weissella* and summarize the commonly used antibiotics in clinical settings where humans were diagnosed and treated/cured of *Weissella* infections.

The *Weissella* genus was first considered a member of the family *Leuconostocaceae* due to their significant shared similarities [1] but later on differentiated into a distinguished genus, which was named ‘*Weissella*’ after the German microbiologist Norbert Weiss [2]. It was reclassified based on the phenotypic and genotypic analysis by Collins in 1993 [3]. The bacteria in this genus are non-spore-forming, generally non-motile, Gram-positive and catalase-negative [4] in nature that exist as either rods or ovoid-shaped cocci [5] belonging to the phylum *Firmicutes* and the family *Lactobacillaceae.* These bacteria are found to thrive in various ecological environments such as soil [6], plants, freshwater lakes [7], spontaneously fermented vegetables, and animal foods [8,9]. They can also be present as commensals on the skin surface and in the saliva and gastrointestinal tract of humans and animals as regular residents. The gastrointestinal tract is particularly thought to be a reservoir for colonization by *Weissella* [10].

## 2. Taxonomy and Sources of Isolation

According to the taxonomy database at The National Center for Biotechnology Information (NCBI, txid46255), as of October 2022, *Weissella* consists of 22 species: *Weissella bombi, Weissella ceti*, *Weissella cibaria, Weissella coleopterorum, Weissella confusa, Weissella diestrammenae*, *Weissella halotolerans*, *Weissella hanii*, *Weissella hellenica*, *Weissella jogaejeotgali*, *Weissella kandleri*, *Weissella koreensis*, *Weissella minor*, *Weissella muntiaci*, *Weissella oryzae*, *Weissella paramesenteroides*, *Weissella sagaensis*, *Weissella salipiscis*, *Weissella soli*, *Weissella thailandensis*, *Weissella uvarum*, and *Weissella viridescens*. However, Teixeira et al. [11] (February 2021) reported that 25 species of *Weissella* have been validated, whereas Fanelli et al. [12] grouped 26 species of *Weissella* into 6 phylogenetic clusters. Outside of the NCBI database, six more species are found in the ‘List of Prokaryotic names with Standing in Nomenclature’ database (https://lpsn.dsmz.de/genus/weissella) accessed on 18 October 2022: *Weissella beninensis, Weissella cryptocerci, Weissella fabalis, Weissella fabaria, Weissella ghanensis,* and *Weissella kimchi.* Twenty-six of these species are validly published under the International Code of Nomenclature (ICNP), except for *Weissella salipiscis*. It is to be noted that when taking into consideration the NCBI and LPSN databases together, 28 species of *Weissella* have been reported (Table 1).

Of the known species of *Weissella*, two *(W. confusa* and *W. cibaria)* have been reported from human or animal clinical infections [13]. However, the metagenome analysis of human fecal samples obtained from IBD patients and controls in our laboratory revealed the presence of several *Weissella* species (Singh et al. unpublished data). All known species of *Weissella* and their varied sources of isolation include: meat (*W. viridescens, W. halotolerans, W. minor*), fermented animal and plant-based food items (*W. confusa, W. jogaejeotgali, W. kimchi, W. hellenica, W. thailandensis, W. koreensis, W. ghanensis, W. sagaensis, W. beninensis, W. fabaria, W. fabalis, W. oryzae, W. hanii,* and *W. salipiscis*), animal/insect sources (*W. ceti*, *W. diestrammenae*, *W. cryptocerci*, *W. bombi*, *W. muntiaci*, and *W. coleopterorum*), wine/wine grapes (*W. paramesenteroides* and *W. uvarum*), soil (*W. soli* and *W. kandleri*), and human samples (*W. cibaria*).

**Table 1:** Summary of sources of most common *Weissella* species.

S. No.	Bacterial Name	Source	Ref.
1	*W. viridescens*	Cured meat	[14]
2	*W. paramesenteroides*	Wine	[15]
3	*W. confusa*	Fermented Greek sausage	[16]
4	*W. kandleri*	Namib desert	[17]
5	*W. halotolerans*	Meat products	[18]
6	*W. minor*	Meat products	[18]
7	*W. hellenica*	Fermented Greek sausage	[16]
8	*W. thailandensis*	Fermented fish	[19]
9	*W. soli*	Soil	[20]
10	*W. cibaria*	Malaysian food and human samples	[21]
11	*W. koreensis*	Kimchi	[22]
12	*W.ghanensis*	Ghanaian cocoa fermentation	[23]
13	*W. beninensis*	Submerged cassava fermentations	[24]
14	*W. fabaria*	Ghanaian cocoa fermentation	[25]
15	*W. ceti*	Beaked whales	[26]
16	*W. fabalis*	Cocoa bean fermentations	[27]
17	*W. oryzae*	Fermented rice grains	[28]
18	*W. diestrammenae*	Gut of a camel cricket	[29]
19	*W. uvarum*	Wine grapes	[30]
20	*W. cryptocerci*	Gut of the insect	[31]
21	*W. bombi*	Bumble bee gut	[32]
22	*W. jogaejeotgali*	Korean fermented seafood	[33]
23	*W. kimchi*	Kimchi	[34]
24	*W. muntiaci*	Feces of Formosan barking deer	[1]
25	*W. sagaensis*	Traditional Chinese yogurt	[35]
26	*W. hanii*	kimchi	[36]
27	*W. salipiscis*	fermented fish	[37]
28	*W. coleopterorum*	Intestine of the diving beetle	[38]

As published in the BV-BRC (Bacterial and Viral Bioinformatics Resource Center) database, as of 19 October 2022, the genome for the genus *Weissella* (Taxonomy Id: 46255) has been reported a total of 448 times, of which the genome for *Weissella cibaria* has been reported the most (n = 168), followed by *Weissella confusa* (n = 128) and *Weissella paramesentroides* (n = 44). The sources of isolation being: human (n = 95), insect (n = 20), avian (n = 9), nonhuman mammal (n = 21), plants, and fermented food sources. The genomes of five species have been reported as isolated from humans: *Weissella cibaria*, *Weissella paramesenteroides, Weissella koreensis,* and *Weissella confusa*. With respect to the genome size, *Weissella* has a smaller pool of genes compared to other fecal commensal bacteria belonging to the genera *Parabacteroides, Bacteroides, Lactobacillus,* and *Pediococcus*. As investigated in our laboratory (Singh et al., unpublished), the genome size and the coding sequence (CDS) of the *Weissella* genus are much smaller than the other fecal bacteria.

## 3. Safety and Virulence Genes

The safety of *W. confusa* has always been a controversial subject due to reports of its isolation from human clinical samples. Although not formally assigned to a risk group by the American Biological Safety Association (ABSA), it has been allocated to Risk Group 1 microorganisms by the German Committee for Biological Agents. The American Type Culture Collection (ATCC) recommends using the strain ATCC 10881^TM^ under biosafety level 1 [7], which makes it unlikely to cause disease in healthy individuals.

Some potential virulence determinants, such as hemolysin, collagen, and adhesin, have been discovered in some of the species of the genus *Weissella* through genome analysis [6], but their role and transferable potential across *Weissella* are still unknown. As in other lactic acid bacteria (LAB), hemolysin genes are universally present in the genus, but their role in pathogenicity remains unproven. The presence of some adhesins may be a desired characteristic in favor of the probiotic potential of *Weissella*. For example, a fibronectin-binding protein (*FbpA*) present in *W. cibaria* strains inhibits the biofilm formed by *S. aureus*, thus being protective against *S. aureus* infections. While there is some evidence to suggest the role of the gut-colonizing potential of *FbpA* in establishing infection in a host, one cannot ignore that the ability for gut-colonization is essential to the probiotic potential of *Weissella,* as demonstrated by Wang et al. [39]. Similarly, mucus-binding proteins play a crucial role in the adhesion of probiotic bacteria to the host gut [40].

It is important to establish the safety of bacteria designed for human consumption to ensure that the organisms are well tolerated and do not pose a health threat when properly administered. To evaluate the safety of these organisms, animal models are typically given higher doses than would be administered to a human. Lyophilized *W. confusa* orally administered to rats at a concentration of 92 × 10^8^ CFU/kg body weight/day for 90 days did not show any evidence of mental or physical ailment when evaluated using a combination of behavioral tests as well as physical examination. Blood cell counts did not show a significant difference in erythrocyte, white blood cell, or lymphocyte concentrations in untreated versus treated rats when controlling for sex [41].

## 4. Opportunistic Infections That Respond to Antibiotics

There have been documented case reports of *Weissella* causing infections in immunocompromised patients, earning it the reputation of an opportunistic pathogen [3]. Reported cases include sepsis, endocarditis, post-operative osteomyelitis, abscess, and meningitis [3,42], among others, as listed in Table 2. Of the listed occurrences of *Weissella* infections, barring a select few, all were successfully resolved with antibiotics. Of the 28 reported cases, 22 were reported to have developed bacteremia, either alone or in conjunction with other infections, such as endocarditis. Fifteen of these people survived upon clinical intervention with antibiotics. At least one reported patient with Crohn’s disease who developed bacteremia had a history of probiotic consumption, which was postulated to be the source of *Weissella’s* introduction into the host body. The probiotic product composition was, however, never investigated.

It is to be noted that the prevalence of *Weissella* in human samples is often under- and over-reported owing to its shared characteristics and features with members of the *Leuconostocaceae* family. Any recently reported increases in isolation can be attributed to associated comorbidities, such as immunodeficiency, history of invasive procedures, organ transplantation, as well as the use of vancomycin, to which *Weissella* is resistant. For most of the healthy population, the source of exposure to *Weissella* is food culture. In available reports, wherever *W. confusa* has been isolated from human tissues, there have been predisposing factors, such as immunocompromised state, prior vancomycin exposure, central venous catheter insertion, history of gastrointestinal procedures/pathologies, and so forth. Due to *Weissella’s* gastrointestinal inhabitance, surgical procedures may lead to the translocation of *Weissella* into the bloodstream. History of vancomycin usage should not be discounted in cases of bacteremia where there has been a possible disruption of the gut microbiome secondary to vancomycin use, allowing for naturally present/ingested *Lactobacilli*/*Weissella* to thrive and overgrow [10].

In the case of a potential infection, it is to be noted that all *Weissella* isolates have been found to be susceptible to antibiotics such as ampicillin, penicillin G, chloramphenicol, erythromycin, doxycycline, minocycline, quinupristin/dalfopristin, gentamicin, and streptomycin [58]. However, some strains exhibit resistance to penicillin [59] and ampicillin [60], and all are resistant to vancomycin [61]. The resistance of *Weissella* is reported to be intrinsic, which may be due to the lack of D-Ala-D-lactate, a target site in their cell wall for vancomycin [42]. Although vancomycin has justifiably been suggested to be a risk factor for *Weissella* infections, their use has been reported in *Weissella* infections in humans that responded to therapy in which other antibiotics were also administered (Table 2). For clinicians, therefore, it is important to be mindful of using vancomycin as the antibiotic of choice in cases of bacteremia that show growth of Gram-positive cocci, not disregarding a possible *Weissella* infection and the in vitro resistance mentioned against vancomycin. There remains, in general, a scarcity of information on the pathogenic pathways of *Weissella* [62].

## 5. Probiotic/Postbiotic Potential and Health Benefits of *Weissella*

There has been a growing interest in studying the different strains of *Weissella* being isolated from diverse ecological environments due to the multitude of prospective pharmacological functions associated with them. Newly isolated *Weissella* strains must meet several criteria to be considered potential probiotic organisms. As per the consensus statement issued by a panel of experts convened by the International Scientific Association for Probiotics and Prebiotics (ISAPP) (October 2013), probiotics are defined as “live microorganisms that, when administered in adequate amounts, confer a health benefit on the host” [63]. In addition to demonstrating a positive effect on the host, a probiotic organism intended for oral ingestion should be able to tolerate conditions in the digestive tract and adhere to the intestinal lining [64]. Lakra et al. [65] sought to evaluate the ability of two newly isolated *Weissella* species to colonize the digestive tract. *W. confusa* MD1 and *W. cibaria* MD2 were both found to be capable of adhering to HT-29 intestinal epithelial cells and mucous surfaces in vitro. Scanning electron microscopy images provided visual confirmation of both strains’ ability to adhere to the intestinal cells in the presence of mucin without damaging the underlying cells [65]. The *W. confusa* strain, Lb. Con, was also evaluated to determine its ability to break down the pesticide chlorpyrifos. The strain showed excellent ability to grow on glucose-free MRS medium supplemented with varying concentrations of chlorpyrifos while being able to degrade 25% of the pesticide. This finding may find its implementation in cases of in vivo and food pesticide toxicity [66].

Recently, much attention has been drawn to the application of postbiotics due to their safety and beneficial advantage in the health and industrial sectors over live bacteria [67,68]. As per the consensus statement issued by a panel of experts convened by ISAPP (2019), postbiotics are defined as a “preparation of inanimate microorganisms and/or their components that confers a health benefit on the host” [69]. These are microbial cells that have been intentionally rendered inactive and may or may not produce metabolites or have cell components that confer the established health benefits. Several research studies have characterized the diversity of metabolites produced by the varied species in the *Weissella* genus [70]. Postbiotics such as bioactive peptides, exopolysaccharides (EPS), enzymes, organic acids, short-chain fatty acids (SCFA), and similar by-products play an important role in the ‘biopreservation’ of food and possess antimicrobial, immunomodulatory, and anti-inflammatory properties [71,72].

### 5.1. Antimicrobial Potential

***Exopolysaccharides***. Of the many metabolites produced by *Weissella*, most previous avenues of research have primarily focused on the diversity of exopolysaccharides (EPSs) secreted by the *Weissella* species. These are a diverse class of macromolecules that help these bacteria execute a variety of functions while protecting them and aiding in general survival [8,73]. The many properties of EPSs include antibacterial, antifungal, antioxidant, and anti-inflammatory functions [9,74], as well as their growth-promoting potential [75,76]. In addition, *Weissella* seems to play an important role in the reduction of a depression-like state [77] and in the strengthening of the gut epithelial barrier [78].

Among *Weissella* species, *W. confusa* is one of the most important EPS producers [11], and different *W. confusa* strains, such as *W. confusa* VP30, XG-3, and KR780676, produce several EPSs with distinct functions. The *W. confusa* strain KR780676 has been shown to produce a galactan EPS that can resist enzymatic degradation in the gastrointestinal tract, as demonstrated in in vitro studies [79]. Interestingly, this EPS also promoted the growth of several probiotic species, including *L. plantarum* and *L. fermentum,* using in vitro screening. When the strain was orally administered to mice, similar effects were observed, as illustrated by an increase in the relative abundance of the probiotic *Lactobacillus* and *Bifidobacterium* species in the stools of these mice [79].

*Weissella cibaria* strains have shown an extensive ability to thwart the population growth of pathogenic microorganisms. Park et al. [75] showed that the EPS produced by *W. cibaria* promoted the growth and the antibacterial activity of a well-established probiotic bacteria, *L. rhamnosus*. The concentration ranges for EPS in the growth media of *L. rhamnosus* defined its antibacterial activity against a range of pathogens. A lower concentration of EPS was sufficient to inhibit the growth of *L. monocytogenes* and *S. aureus*, thereby demonstrating a higher antibacterial activity against these bacteria, whereas the growth of *B. cereus* and *E. coli* was inhibited at higher concentrations of EPS. In another study, a higher yield of EPS was observed in the *W. cibaria* strain W27 (isolated from kimchi) when supplemented with sucrose. This also improved the bacteriocinogenic potential of the bacteria by inducing its surface property [80]. The assimilation of sucrose helped enable the hydrophobic nature of W27 that ultimately contributed to the enhanced antibacterial activity as observed against *S. aureus*, *B. cereus,* and *E. coli.* Another study by Yeu et al. [81] illustrated the inhibitory effect that the EPS derived from *W. cibaria EIR/P2* has on biofilm formation by the pathogens responsible for upper respiratory tract infections: *S. aureus, Streptococcus pyogenes, Streptococcus pneumoniae,* and *Moraxella catarrhalis*. With respect to removal of preformed biofilms, the best effect was elucidated against *S. aureus,* while no effect against *S. pneumoniae* was observed. In a separate study, the strain *W. cibaria* JW 15 was shown to exhibit an anti-biofilm effect against *S. aureus*, *L. monocytogenes*, *S. enterica,* and *S. typhi* [82,83], which are all known to possess pathogenic potential capable of causing serious infections.

***Bacteriocins***. Probiotic bacteria also show bactericidal or bacteriostatic activity though the synthesis of small ribosomal peptides called bacteriocins [84]. Only six purified bacteriocins have been reported to be produced by *W. cibaria, W. paramesenteroides,* and *W. hellenica* [85]. Srionnual et al. [86] were the first to report a unique bacteriocin called ‘*weissellicin 110’* produced by the *W. cibaria* strain, 110 (isolated from a Thai fermented fish product). However, the full amino acid sequence information of *w110* was not calculated by the author at the time. The bacteriocin *w110* showed narrow-spectrum antibacterial activity against some Gram-positive bacteria but did not inhibit the growth of food-borne pathogen *L. monocytogenes*. In 2017, Li et al. [87] deduced the full amino acid sequence of *w110* through whole-genome sequencing and classified it into class IId due to the presence of unique genes and for having a 21-amino-acid N-terminal leader peptide. Teixeira et al. reported similar narrow-spectrum antibacterial activity against LAB in another strain of *W. cibaria*, *W25* [88]. Based on AntiSMASH analysis, the authors proposed the synthesis of two different types of bacteriocins in *W. cibaria* W25 being produced by the translation of lassopeptide (MicJ25) and RiPP-like bacteriocin_IIc genes, but the identification of these bacteriocin-producing genes was not confirmed by the Bagel 4 software (http://bagel4.molgenrug.nl/, accessed on 18 October 2022).

Another novel bacteriocin named ‘*weissellicin D’* was reported to be synthesized by *W. hellenica* strain D1501 (isolated from fermented meat) [89]. The thermostable *‘weissellicin D’* exhibited a broad range of antibacterial activity against many food-borne pathogens, such as *E. coli*, *S. aureus,* and *L. monocytogenes*. It was also found to inhibit the growth of yeasts and molds that included *Candida albicans, Debaryomyces*, *Mucor*, *Saccharomyces cerevisiae,* and *Kluyveromyces marxianus*. However, possibly due to its autoimmunity, *weissellicin D* did not affect the growth activity of its own producer strain. Later, Chen et al. [90] successfully exploited the antagonistic activity of *W. hellenica* D1501 to improve the shelf life of tofu by simultaneously co-culturing it with spoilage bacteria such as *E. coli*, *S. aureus,* and *K. gibsonii* in soymilk.

Bacteriocin ‘*weissellicin A*’ was identified and characterized from the strain *W. paramesenteroides* DX. It was heat-resistant and showed activity against a range of Gram-positive bacteria [91]. The bacteriocin was classified into class II and predicted to affect the integrity of plasma membranes of pathogens, causing an efflux of required nutritious cellular metabolites, thereby resulting in cell death. The thermostable and acid-resistant potential of this bacteriocin can be used in the preservation of acidic foods at an industrial scale. Another industrially important bacteriocin, ‘*weissellicin L’,* produced by *W. hellenica* was reported by Leong et al. [92], and the nucleotide characterization performed by Chen et al. [93] a year later declared it to be unique. The bacteriocin *weissellicin L* strongly inhibits *L*. *monocytogenes* and, therefore, can be used in the biopreservation of chilled food, which is mostly contaminated by this spoilage pathogen.

The production of bacteriocins by any given *Weissella* strain depends on the nutrient composition and availability of vitamins in the culture medium where the bacterium grows. Isolated from Japanese pickles, *W. hellenica*, QU 13, as observed by Masuda et al. [94], was interestingly found to produce multiple bacteriocins. The authors discovered that QU13 produced two different bacteriocins, named *‘weissellicin Y’* and *‘weissellicin M’,* based on their nutritional preferences. While *weissellicin Y* is produced in the MRS media, *weissellicin M* is produced in the thiamine-rich media, APT. The vitamin, while accelerating the growth of *W. hellenica* QU 13 on the one hand, reduced the synthesis of *weissellicin Y* on the other. However, the production of *weissellicin M* was not affected by this transition at all. The mechanism of production of these bacteriocins is still not fully elucidated. Both *weissellicin Y* and *weissellicin M* possess broad antimicrobial spectra specifically targeted against *B. coagulans*. Between the two, *weissellicin M* showed comparatively higher antibacterial activity, as well as greater acid and thermal stability when compared to *weissellicin Y*.

Among other strains of *Weissella*, *W. confusa* Cys2-2 (isolated from ginger) was observed to produce a bacteriocin that showed bactericidal activity against the Gram-negative enterics, *E. coli, Salmonella,* and *Shigella* [95]. The Cys2-2 bacteriocin exerted this effect by altering the membrane integrity of target cells. Similar broad-spectrum antibacterial activity was reported by Goh and Philip [96] in another *W. confusa* strain, A3 (isolated from a dairy source), against bacteria such as *B. cereus*, *E. coli*, *P. aeruginosa,* and *M. luteus*. No inhibitory effect on the growth of *S. aureus* was noted. Yet another strain, *W. confusa* GCC_19R1, was found to have antibacterial activity against the Gram-negative bacteria *Stenotrophomonas maltophilia*, *Acinetobacter johnsonii*, *Achromobacter spanius,* and *Cedecea davisae* [85]. The bacteriocin synthesized by the *W. confusa* strain, PL9001, exhibits antagonistic activity against the pathogen *Helicobacter pylori* and can be used to treat H. pylori-induced gastritis and gastric ulcers [97,98]. *Weissella paramesenteroides* DFR-8 (isolated from cucumber) is reported to produce a thermostable bacteriocin that shows a broad-spectrum antimicrobial effect against both Gram-positive and -negative organisms [99]. In another study, the authors Pal and Ramana [99] reported the production of non-bacteriocin antimicrobial components from the same strain of *Weissella* that proved effective against Gram-negative bacteria.

BLIS: Apart from bacteriocins, some species of *Weissella* have also been found to produce ‘bacteriocin-like inhibitory substance’ (BLIS), a bacteriocin that has neither been fully characterized nor purified. For instance, *W. confusa MBF8-1* isolated from fermented soybean showed BLIS activity against *Leuc. mesenteroide* and other closely related species [100]. Encoded by a large plasmid, pWcMBF8-1, the strain *MBF8-1* produced a BLIS called *‘weissellicin MBF*’.

Other metabolites/mechanisms: Some of the other miscellaneous mechanisms through which the *Weissella* spp. exhibit antimicrobial effects include: the production of hydrogen peroxide, organic acids (lactic, acetic, and citric acids), fatty acids, and specific proteins, e.g., N-acetylmuramidase. Lim et al. [101] successfully explored the antimicrobial activity of *W. cibaria*, CMU, against oral pathogens, possibly due to acid and hydrogen peroxide. Another strain of *W. cibaria*, KY10, isolated from shrimp gut, was shown to have bactericidal activity against *Vibrio parahaemolyticus* T.11 through the mechanism of organic acid release [102]. In another study, Dey et al. [103] examined the antibacterial activity of *W. confusa* DD_A7 isolated from kimchi and found that the DD-A7 strains trigger the oxidative stress to inhibit the growth of extended-spectrum β-lactamase (ESBL)-positive *E. coli*, which are emerging pathogens. *Weissella* also acts as an anti-mycobacterial, possibly due to its obligate heterofermentative nature, which makes it unique and prominent among other LAB. The authors emphasized that *Weissella* generates lactate and ethanol as by-products at equivalent concentrations that could have a stronger anti-mycobacterial effect than lactate alone [104].

### 5.2. Immunomodulatory and Anticancer Potential

Different species within the *Weissella* genus have been investigated for their role as potential probiotic organisms. There are several mechanisms through which such species may provide a positive health effect for the host. Probiotic organisms can modulate the immune system, reducing excess or unwarranted inflammation while simultaneously priming the host’s defenses and immune system against pathogenic organisms. The multifaceted nature through which probiotic organisms interact with the host’s immune system is still incompletely understood, although it is known that these organisms can change the way immune cells respond to identical stimuli.

One mechanism through which some species in the *Weissella* genus exert anti-inflammatory effects is through the modulation of the NF-κB-mediated signaling pathway. *W. confusa* downregulates the expression of the iNOS gene responsible for regulating the production of nitric oxide (NO), a proinflammatory mediator produced from L-arginine [103]. *W. confusa* also metabolizes and uses arginine for its own growth, consequently further decreasing the NO levels in the body. Using a larval zebrafish model system, Dey and Kang [105] demonstrated that the EPS produced by *W. confusa* can reduce inflammation caused by *E. coli*-derived Shiga toxin. This finding is supported by previous research using a murine model system that found evidence that the EPS produced by *W. confusa* can modulate the immune system by increasing the level of Immunoglobulin A, the absence of which leads to intestinal inflammation [106].

Within the intestine, the intactness of the intestinal epithelial barrier is an important marker for gut health. Reduced adhesion between adjacent intestinal epithelial cells, termed “leaky gut”, causes excessive permeability of the intestine and disruption of intestinal homeostasis, resulting in aberrant immune activation [107]. Restoration and maintenance of barrier function is an important trait to be assessed for in orally administered probiotics. One strain of *W. cibaria* named MW01, isolated from Chinese sauerkraut, was found to restore the barrier integrity of Caco-2 intestinal epithelial cells (IECs) after barrier dysfunction was induced by LPS [106]. The restoration of barrier integrity was accomplished through the inhibition of the nuclear translation of NF-κB and the subsequent blocking of the MLCK (mitogen-activated protein kinases) pathway. This led to the upregulation of the genes encoding for tight junction proteins (TJP), as well as increased TJP protein levels, and reduced release of proinflammatory cytokines such as IL-6, IL-8, and TNF-α [106]. This finding was corroborated by Silva et al. [108] when they analyzed the cell-free supernatant after administering *W. cibaria* CIATEJ BI–48.1 to confluent HT-29 monolayers. Several short-chain fatty acids (SFCAs) were detected, including acetic and butyric acids. SFCAs are important metabolites in the human digestive tract, where upward of 90% of butyrate in the intestinal lumen is metabolized to meet the energy needs of the colonocytes [109]. SCFAs such as acetic and butyric acid are also considered important modulators of inflammation in the colon and promote the upregulation of TJPs in the small intestine [110]

Probiotics in general can help reverse the gut dysbiosis implicated in the pathogenesis of several inflammatory and auto-immune conditions as well as cancers, and thereby help prevent these conditions or attenuate their severity. Certain strains of *Weissella* are being investigated as an adjuvant to conventional treatment in IBD. Various in vitro and animal models have explored this. For example, *W. paramesenteroides* WpK4 was able to reduce the disease activity index (DAI) as well as repair some of the mucosal damage in mice models with DSS-induced colitis. The bacteria also helped reduce the production of proinflammatory markers such as TNF- α, NO, IL-1β, and IL-6. As a consequence of this immunomodulation, the colitis mice also demonstrated reduced anxiety and depression-associated behavior [77]. The *W. confusa* strain F213 was shown to maintain the transepidermal resistance in an in vitro intestinal cell model employing Caco-2 cells where hydrogen peroxide was used to induce IBD. It also decreased intestinal permeability as well as helped maintain tight junctions, the disruption of which is seen in IBD [111]. The EPS purified from milk fermented with *W. confusa* VP30 was found to have a remarkable laxative effect on the constipated rat model and can be used to relieve constipation issues in humans [112].

Some strains of *Weissella,* such as *W. cibaria,* demonstrate anticancer effects against colorectal cancer by suppressing cell growth. Cha et al. [113] have a patent establishing the anticancer effect of *W. cibaria* against colorectal cancer cells by selectively suppressing the growth of cancer cells as opposed to normal cells. A review by Kwak et al. [114] investigated the benefits of kimchi LAB (*W. cibaria* and *L. plantarum*) against cancer and derived the same conclusion about *W. cibaria* [114]. The anti-proliferative activity of *W. cibaria* against cancer cells such as HeLa and Caco-2 has been confirmed in other studies as well [112,115,116]. A unique single-center study by Kwon et al. investigated the role of probiotic usage in post-operative cancer patients and noticed an increase in the populations of several beneficial bacteria that also included a very significant increase in the *Weissella* bacteria (a rise from 0.096% to 0.361%, *p* < 0.004) after 8 weeks of probiotic supplementation. This increase correlated with the improved quality of life in these patients [117]. Recently, Amer et al. [118] explored the anticancer effect of exopolysaccharides nanoparticles (EPS-NPs) produced by *W. paramesenteroides* MN2C2 against breast MCF-7, colon Caco-2, and liver HepG-2 malignant cells due to its potent antioxidant potential. These research findings could be an innovative alternative approach in cancer treatment, minimizing the use of prolonged conventional therapy and its adverse side effects.

*Weissella cibaria* has also been acknowledged for its ability to prime the immune system in immunocompromised hosts. Park and Lee [75] investigated the role of *W. cibaria* JW15 as a complementary treatment for counteracting the weakened immune system as a consequence of chemotherapy. Oral administration of the bacteria to immunosuppressed (secondary to cyclophosphamide) BALB/c mice resulted in increased splenocyte proliferation and elevated white blood cell counts (WBC). Additionally, *W. cibaria* JW15 was able to increase the production of TNFα, IL-1β, and IL-6 when challenged with purified lipopolysaccharide (LPS). Together, these results demonstrate the capacity of the *W. cibaria* JW15 strain to enhance the innate immune response in immunocompromised mice. Furthermore, no evidence of toxicity or bacteriemia because of the administered bacteria was observed in the treated mice despite their weakened immune system. Other studies by the same set of researchers in aged mice found similarly increased populations of red blood cells, WBS, and splenocytes along with elevated levels of cytokines IL-6 and IFN-γ [119]. When compared to the commercial strain of *L. rhamnosus* GG, the Weissella strain JW15 had higher immune-stimulating activity. In a nutshell, JW15′s use as a probiotic can help augment the immune response of the host [120]. In contrast to the aforementioned studies, the study by Seok et al. observed the suppression of the immune system in an in vitro murine model and illustrated the reduced expression of proinflammatory cytokines IL-6, IL-1β, and TNFα through inhibited activation of the NF-κB pathway in murine RAW 264.7 macrophages exposed to LPS. In addition, the administration of heat-killed *W. cibaria* JW15 resulted in the decreased production of nitric oxide and prostaglandin E2 [121].

### 5.3. Dental and Skin Health

Many of the traits that allow organisms such as *W. cibaria* and *W. confusa* to play a beneficial role in the gut also allow these organisms to be advantageous to other sites in the body that are susceptible to the same environmental challenges as the intestinal lumen. Particularly, *W. cibaria* has been shown to display probiotic effects in other body sites in animal studies. Using an induced-periodontitis mouse model, Kim et al. demonstrated that *W. cibaria* CMU could reduce the severity of periodontitis in a dose-dependent manner [106]. Mice treated with this strain displayed reduced alveolar bone loss and lower levels of the pathogenic *Porphyromonas gingivalis*. The authors posit that *W. cibaria* CMU has the capacity to adhere to saliva-coated surfaces in the mouth, as well as produce antimicrobial compounds that reduce the growth of pathogenic species that are responsible for the development of periodontitis. In a similar set of experiments, *W. cibaria* CMU was found to inhibit the co-aggregation of another oral pathogen, *Fusobacterium nucleatum,* which is also known to contribute to the development of periodontitis [122]. Additionally, this strain of *W. cibaria* reduced IL-6 and IL-8 secretion by oral epithelial cells previously challenged with *F. nucleatum.* A study by Kibar et al. into *W. cibaria* EIR/P-2 (isolated from bee pollen) displayed its antibacterial activity against *Streptococcus mutans*, a bacterium notorious for causing tooth decay and caries. The dextran derived from the EPS also had proliferative activity, which, coupled with the antimicrobial/antibiofilm functions, may find its application in the field of dentistry as an agent of ‘periodontal healing and regeneration’ [82].

*Weissella* has been found to have applications in the skin industry as a prospective treatment for many skin conditions. The WIKIM28 strain of *W. cibaria* has been shown to benefit patients with atopic dermatitis [79]. The authors used an induced-dermatitis model made by exposing mice to 2,4-dinitrochlorobenzene, which created skin lesions and thickened the epidermal layer mimicking dermatitis in humans. Oral administration of *W. cibaria* WIKIM28 resulted in a reduction of symptoms in treated mice, as observed through improved histological scoring of skin sections [79]. Additionally, the treated mice displayed an increased ratio of differentiated CD4^+^CD25^+^Foxp3^+^ regulatory T cells (Tregs), along with a corresponding increase in IL-10 in polyclonal mesenteric lymphocytes, which is associated with a suppressed immune response. Taken together, these results indicate that *W. cibaria* WIKIM28 is capable of reducing inflammation in the skin through interactions between the spleen, lymphatic system, and the intestine.

### 5.4. Anti-Obesity

Probiotic bacteria help maintain metabolic homeostasis by producing active metabolites [123]. *Weissella* spp. such as *W. koreensis* (isolated from kimchi) have been reported to exhibit anti-obesity effects by regulating lipid metabolism. A study by Moon et al. [124] found that *W. koreensis* OK1-6 metabolized arginine into L-ornithine, a non-protein amino acid that down-regulated the expression of adipocyte-specific genes C/EBPα, aP2, SREBP1, and fatty acid synthase (FAS) in 3T3-L1 cells lines, thus preventing the accumulation of intracellular lipid and triglyceride inside the cells [125].

Choi et al. [126] reported the anti-obesity effect of *W. cibaria* MG5285 in mice with high-fat diet (HFD)-induced obesity. The study showed a significantly reduced expression of lipogenic proteins, including peroxisome proliferator-activated receptor γ (PPAR-γ), CCAAT/enhancer-binding protein α, FAS, and adipocyte-protein 2. In addition, sterol regulatory element-binding protein 1-c and its downstream protein FAS in the liver tissue were significantly decreased. These strains attenuated fat accumulation in the liver by upregulating the phosphorylation of AMP-activated protein kinase and acetyl-CoA carboxylase in the HFD-fed mice. Another study evaluated the anti-adipogenic capacity of whey that had been ‘biotransformed’ by *Weissella cibaria* in 3T3-L1 adipocytes and found a disruption in the intracellular signaling pathways responsible for the expression of obesity-associated genes and transcription factors, such as PPAR-γ, resulting in reduced accumulation of triglycerides and lipids inside the 3T3-L1 cells [127].

## 6. Starter Culture

*Weissella* spp. have tremendous functional and technological potential, which can improve the safety and nutritional and sensory characteristics of food. They enhance the flavor and improve the texture of food by producing beneficial metabolites such as organic acids, short-chain fatty acids, and esters during food fermentation [128,129,130]. They have been used as a starter culture in various ethnic foods in Europe and Asia [58]. For instance, *W. cibaria* and *W. confusa* have been studied in the fermentation of bread. These starter strains have been observed to enhance the production of functional components and rheological attributes of bread by eliminating the need for baker’s yeast [131] and also improving the softness [132] and texture of gluten-free bread [133]. In addition, *W. cibaria* has been studied in the manufacturing of functional bread fortified with riboflavin [134]. The starter culture *Weissella viridescens* F2 was found to reduce the accumulation of biogenic amines and to improve the quality of Roucha during fermentation [135]. An ornithine-producing strain, *W. koreensis DB1*, isolated from kimchi, was studied in the fermentation of rice bran and found to have a significant effect on the organoleptic properties of rice. Such starter cultures offer a dual role by contributing to food functionality through its fermentation.

Despite having probiotic and outstanding starter culture potential, the genus has not yet been permitted for commercial use in the United States or the European Union nor acknowledged as a part of the International Dairy Federation Inventory [7,136] due to the lack of GRAS (Generally Recognized as Safe) status. The vast diversity of this genus and the deficiency of scientific literature could be possible reasons for not being granted the GRAS status by the FDA (Food and Drug Administration) or by the EFSA (European Food Safety Authority). The genus is also often classified as opportunistic pathogens, which seems to limit its application as potentially beneficial probiotics in the food industry despite being potentially powerful starters for food fermentation [85,137].

## 7. Conclusions

The *Weissella* genus comprises environmentally omnipresent bacteria that also happen to exist as commensals in the gastrointestinal tract of healthy vertebrates. In particular, the species *W. confusa* and *W. cibaria* have become subjects of extensive research in recent times, most of which involve an in-depth evaluation of their myriad health benefits as probiotics. The other end of the research looks into the cases of proposed opportunistic pathogenicity in people with underlying medical conditions. To date, the scales tip in favor of the many health benefits attributable to the use of these species, whereas cases of bacteremia, endocarditis, and meningitis remain low, with infections being seen in already-compromised hosts with predisposing factors, such as immunocompromised status, hospital procedures, and medical comorbidities, to name a few. Members of *Weissella* species are not generally known to infect healthy populations. All reported cases have been successfully treated with a variety of antimicrobials that the *Weissella* species remain sensitive to. The use of vancomycin is discouraged, however, owing to extensive resistance seen in this genus against the antibiotic. *Weissella’s* remarkable antimicrobial and anti-inflammatory nature speak to its probiotic potential. With significant research backing the use of *Weissella*, the genus may come to be recognized as an important probiotic in the near future, with applications spanning across industries.

## Figures and Tables

**Table 2:** Antimicrobials and clinical infections with *Weissella confusa:* humans respond very well to antibiotics.

Age, Sex	Underlying Conditions	Clinical Infection	Treatment	Outcome	Ref.
12, F	Gastrostomy	Bacteremia	Cephalosporin	Cured	[43]
71, M	Cecal carcinoma	Bacteremia	Cephalosporin	Cured	[43]
-	-	Organ colonization	Ampicillin	Cured	[44]
49, M	None	Abscess infection	Cephalothin	Cured	[45]
-	-	Organ colonization	Ampicillin	Cured	[46]
46, M	Abdominal aortic dissection repair, coronary artery bypass grafting, parenteral nutrition	Bacteremia	Piperacillin-tazobactam	Cured	[47]
49, M	Alcohol abuse history, treatment with corticosteroids	Endocarditis, Bacteremia	None	Fatal	[48]
65, M	Aortic insufficiency	Infective endocarditis	Penicillin G, gentamicin, moxifloxacin, cefoperazone	Cured	[49]
56.6, 6F, and 4M	Malignancy (4), chronic steroid use (3), chemotherapy (3), abdominal surgery (4), polymicrobial infection (5), central catheter (6)	Bacteremia	Vancomycin, ceftazidime, ampicillin-sulbactam, amoxicillin-clavulanate, gentamicin, ciprofloxacin, and trimethoprim-sulfamethoxazole	Cured (4), Fatal (6)	[50]
34, M	Hematopoietic stem cell transplant recipient	Bacteremia	Vancomycin, aztreonam, and daptomycin	Cured	[51]
58, M	Severe burns, polymicrobial infection, central catheter	Bacteremia	Vancomycin, imipenem, and daptomycin	Cured	[51]
54, M	Hepatocellular carcinoma, Liver transplant, hepatic artery thrombosis, diabetes	Bacteremia	Metronidazole and levofloxacin	Cured	[52]
48, M	Gastroesophageal adenocarcinoma	Bacteremia	Cefoperazone-sulbactam Metronidazole	Cured	[53]
60, F	Hypertension, aortic intramural hematoma	Bacteremia	Teicoplanin and piperacillin-tazobactam	Cured	[54]
94, F	Osteoarthritis, total knee arthroplasty	Prosthetic joint	Levofloxacin	Cured	[55]
63, F	Crohn’s disease with gastrointestinal strictures, central venous catheter	Bacteremia	Piperacillin/tazobactam	Cured	[56]
14, M	Medulloblastoma, surgery, chemo and radiotherapy, polymicrobial infection	Bacteremia	Clindamycin, amikacin	Cured	[57]
78, M	Immunodeficiency	Meningitis	Ampicillin		[3]
25, M	Crohn’s disease, short bowel syndrome, intestinal failure	Bacteremia	Meropenem, metronidazole, and cefuroxime	Cured	[10]
